# RPFdb: a database for genome wide information of translated mRNA generated from ribosome profiling

**DOI:** 10.1093/nar/gkv972

**Published:** 2015-10-03

**Authors:** Shang-Qian Xie, Peng Nie, Yan Wang, Hongwei Wang, Hongyu Li, Zhilong Yang, Yizhi Liu, Jian Ren, Zhi Xie

**Affiliations:** 1 State Key Laboratory of Ophthalmology, Zhongshan Ophthalmic Center, Sun Yat-sen University, Guangzhou 510060, China; 2 Scientific Center for Precision Medicine, Sun Yat-sen University, Guangzhou 510000, China; 3 State Key Laboratory of Biocontrol, School of Life Sciences, Sun Yat-sen University, Guangzhou, Guangdong 510275, China; 4 Division of Biology, Kansas State University, Manhattan, KS 66506, USA

## Abstract

Translational control is crucial in the regulation of gene expression and deregulation of translation is associated with a wide range of cancers and human diseases. Ribosome profiling is a technique that provides genome wide information of mRNA in translation based on deep sequencing of ribosome protected mRNA fragments (RPF). RPFdb is a comprehensive resource for hosting, analyzing and visualizing RPF data, available at www.rpfdb.org or http://sysbio.sysu.edu.cn/rpfdb/index.html. The current version of database contains 777 samples from 82 studies in 8 species, processed and reanalyzed by a unified pipeline. There are two ways to query the database: by keywords of studies or by genes. The outputs are presented in three levels. (i) Study level: including meta information of studies and reprocessed data for gene expression of translated mRNAs; (ii) Sample level: including global perspective of translated mRNA and a list of the most translated mRNA of each sample from a study; (iii) Gene level: including normalized sequence counts of translated mRNA on different genomic location of a gene from multiple samples and studies. To explore rich information provided by RPF, RPFdb also provides a genome browser to query and visualize context-specific translated mRNA. Overall our database provides a simple way to search, analyze, compare, visualize and download RPF data sets.

## INTRODUCTION

Translational control is crucial in the regulation of gene expression. Deregulation of translation is associated with a wide range of cancers and human diseases (1). For example, hereditary hyperferritinaemia-cataract syndrome (HHCS) is an autosomal dominant disorder caused by mutations in the iron-response elements of ferritin. The mutation causes increased translation of ferritin mRNA and, hence, elevated serum levels of ferritin. This leads to nuclear cataract, an eye disease that eventually progresses to total blindness (2). Therefore, accurate measurement of translated mRNA is invaluable to better understand cellular functions and human diseases.

Nuclease footprinting is a conventional way to determine ribosome positions on mRNA, where the 28–30 nucleotides of mRNAs protected by a ribosome indicate translated mRNA (3,4). Ribosome profiling is a recently developed high-throughput strategy based on deep sequencing of ribosome-protected mRNA fragments (RPF) (5,6), that provides genome-wide information of mRNA in translation. Since its inception in 2009, RPF technique has been utilized in a range of studies in both prokaryotic and eukaryotic organisms and the number of studies increases rapidly every year (4,6–8).

To date, GWIPS-viz is the only database specifically designed for RPF data sets (9). GWIPS-viz provides a very valuable online genome browser to view the coverage and distribution of RPF reads. Currently it hosts RPF and mRNA data sets from 45 studies. Another relevant database is TISdb that is based on the recently developed Global Translation Initiation sequencing (GTI-seq) technology which provides global mapping of translation initiation codons (10). TISdb provides tools to search for translation initiation sites and the associated open reading frames (ORFs) based on multiple GTI-seq datasets. Since the numbers of studies using RPF technique have been growing significantly in the recent years, there is a strong need for an integrated database that facilitates the exploration of data from these studies. Furthermore, in addition to visualization of RPF reads, there is also an emerging database demand both for hosting the meta information of the studies but also for in-depth analysis of studies in a consistent way.

**Figure 1.**
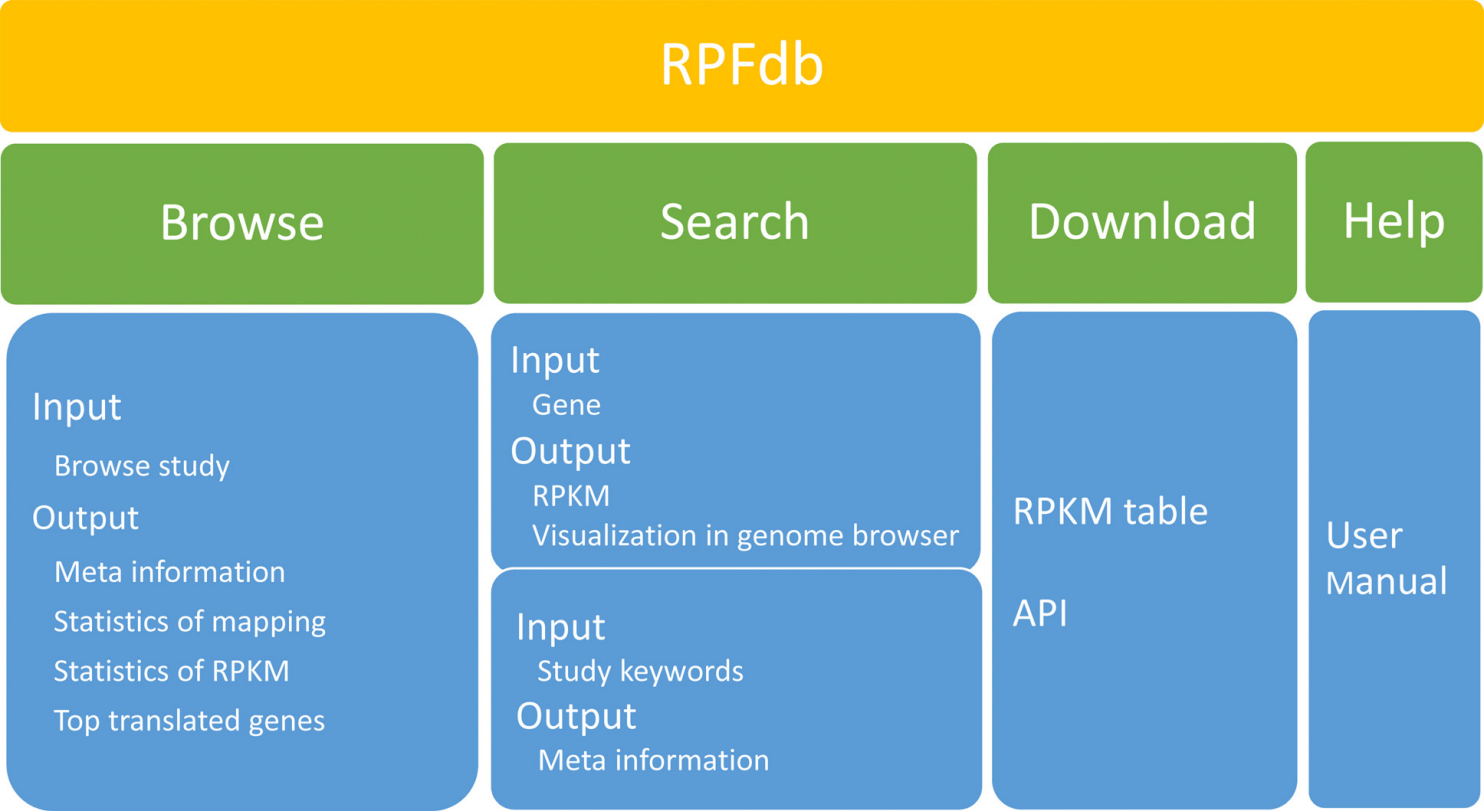
Structure and contents of RPFdb.

Herein, we present RPFdb, a comprehensive resource for hosting, analyzing and visualizing RPF data sets, available at www.rpfdb.org or http://sysbio.sysu.edu.cn/rpfdb/index.html. The current version of database contains 777 samples from 82 studies in 8 species, reprocessed by a unified pipeline. The main functions of the database include Browse, Search and Download, summarized in Figure 1. There are two ways to query the database: by keywords of studies or by genes. The outputs are presented in three levels. (i) Study level: including meta information of studies and reprocessed data for gene expression of translated mRNAs; (ii) Sample level: including global perspective of translated mRNA and a list of the most translated mRNA of each sample from a study; (iii) Gene level: including normalized sequence counts of translated mRNA on different genomic location of a gene from multiple samples and studies. To explore the rich information provided by RPF, RPFdb also provides a genome browser to query and visualize context-specific translated mRNA. Overall our database provides a simple way to search, analyze, compare, visualize and download RPF data sets.

## MATERIALS AND METHODS

### Data sources

**Figure 2.**
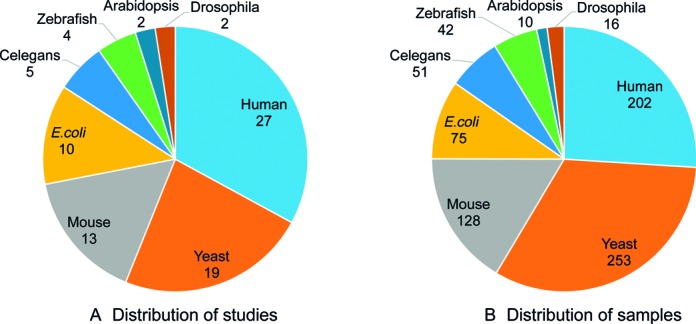
Distribution of studies and samples in species

The RPF sequencing data were collected from Gene Expression Omnibus (GEO) and Short Read Archive (SRA) databases. The current version contains 777 samples from 82 studies in 8 species: Arabidopsis, C. elegans, Drosophila, E. coli, Human, Mouse, Yeast and Zebrafish. Figure 2 shows the distribution of studies and samples by species. Human and yeast are the two species that were most studied.

### Data processing

**Figure 3.**
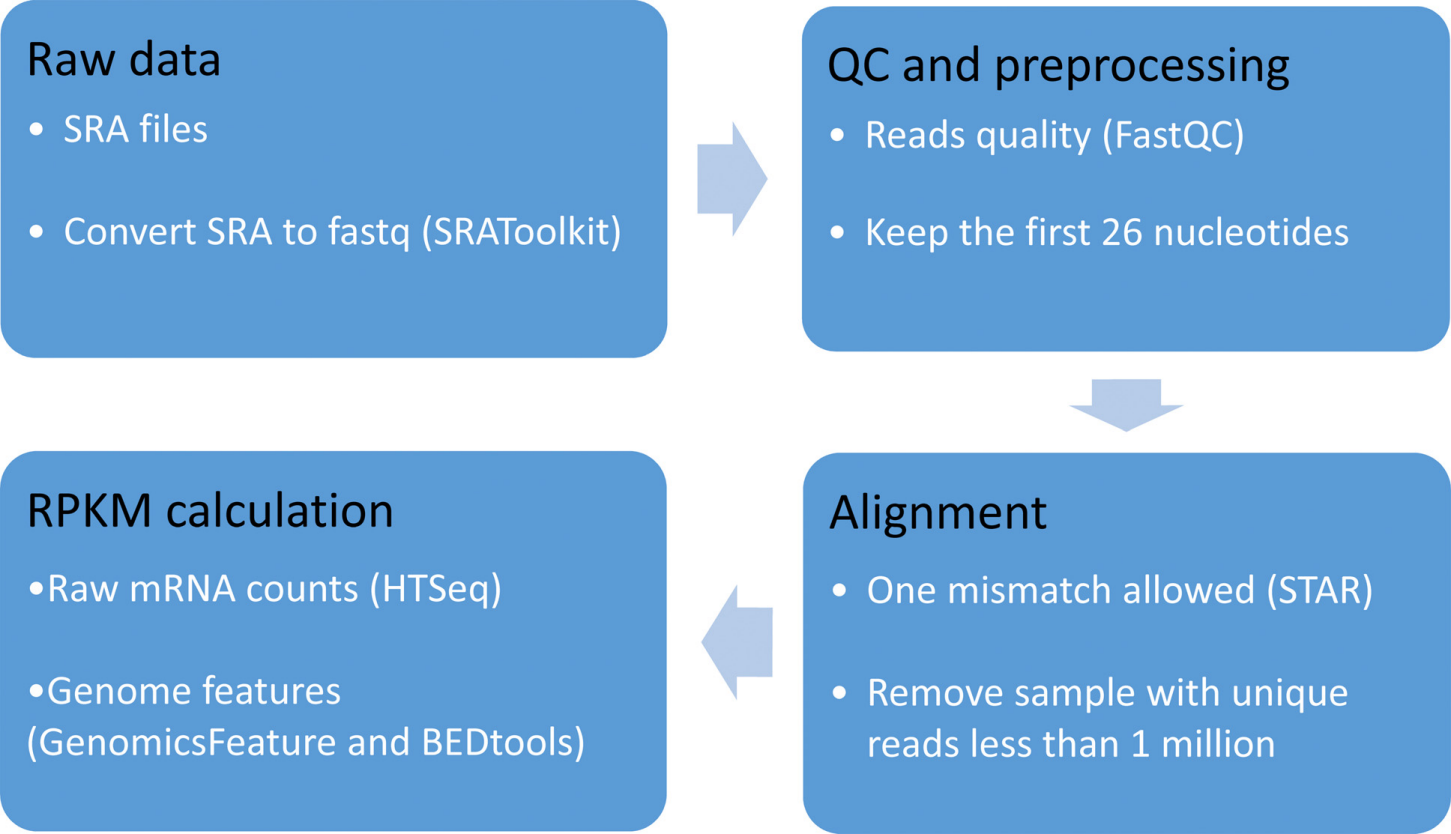
Data processing and RPKM calculation

The pipeline for data processing is summarized in Figure 3. Specifically, SRAToolkit v2.4.3 (http://www.ncbi.nlm.nih.gov/Traces/sra/?view=software) was used to convert sra files to fastq format. And FastQC v0.11.2 (http://www.bioinformatics.babraham.ac.uk/projects/fastqc) was used to access the base quality of raw data. Since ribosome encloses a ∼30 nucleotides of mRNA and the 3′ sequences are usually linkers (5), we selected the first 26 nucleotides of the sequences for the subsequent alignment against reference genome as described in the literature (11). STAR v2.4.0i was used for alignment where one mismatch was allowed and multiple alignments were accepted (12). Supplemental Table S1 shows reference genome and related annotation files of eight species. We removed any sample if the number of its uniquely mapped reads is less than 1 million.

### Data analysis

RNA–SeQC was used to generate two types of statistics of mapped reads: (i) the numbers of mapped and unmapped reads of each sample and (ii) the mapped ratio in exonic, intronic and intergenic regions of each sample (13). Reads Per Kilobase per Million mapped reads (RPKM) was used to measure RPF abundance, as defined below (14).

\begin{equation*} RPKM = r_f \times 10^9 /(R \times fl_f ), \end{equation*}

where *R* is the total mapped reads in all genes, *fl_f_* is the feature length and *r_f_* is the raw mRNA counts.

The raw mRNA counts was calculated by HTseq-count v0.6.1p1 (15). Exonic loci of CDS and exon, were retrieved directly from annotation file against reference genome (Supplemental Table S1), and exonic loci of 3′ UTR and 5′ UTR were extracted by R package GenomicFeatures (16). If an exonic locus was classified as UTR for one transcript and as CDS for another transcript of the same gene, UTR or CDS region for each transcript was annotated separately, and the same exon was counted as both UTR and CDS. The feature length from multiple transcripts of the gene were merged.

### Database implementation

The database was implemented by PHP, MySQL and JavaScript. The study and gene information were stored and queried by MySQL and PHP. The JavaScript JQuery and D3.js library were used for producing dynamic and interactive data visualization in the web browser. In addition, we integrated JBrowse in our database for visualizing context-specific translated mRNA intuitively. And the aligned RPF sequences in each species against their reference and annotation information are hosted in the genome browser.

## RESULTS

### Usage and access

The RPFdb includes Home, Browse, Search, Download and Help pages.

#### Search

This page provides two ways to query the database. (i) Search gene: by selecting a species and entering a gene symbol or Ensembl ID in the search box of the search page (also appears in the home page), the output shows the genome information of this gene from all the samples for the selected species, including RPKM of the gene and RPKM of the 5′ UTR, CDS and 3′ UTR regions. The users can sort the table by clicking the column names. The users may also use a search box on the output page to filter the results. A snapshot of output of ‘Search gene’ is shown in Supplemental Figure S1. The JBrowse icon provides hyperlink to a genome browser, which is described in the next section. (ii) Search study: users can search studies by keywords. This feature is useful to retrieve data set from the curated studies in the database. A snapshot of output of ‘Search study’ is shown in Supplemental Figure S2.

#### Genome browser

To explore the distribution of RPF reads for a given gene, RPFdb provides a genome browser to query and visualize context-specific translated mRNA. A snapshot of an example of ‘Genome browser’ is shown in Supplemental Figure S1. The annotated gene track and reference are displayed on the top of browser. RPF reads of the selected gene are shown at the bottom. If users select multiple samples, they can easily compare reads distribution on the genome.

#### Browse

For each study, this page displays: (i) Meta information of the study, including abstract of study, tissue or cell source, treatment for RPF experiment and reference genome for alignment (Supplemental Figure S3); (ii) The top 200 most translated genes. In addition, the whole RPKM table of the study is also downloadable (Supplemental Figure S4); (iii) Plots showing overall statistics of each sample, including the numbers and fraction of mapped and unmapped reads in each sample, and statistics of RPKM on different genomic regions of each sample, including 5′ UTR, CDS, 3′ UTR and gene (Supplemental Figures S3 and S4).

#### Download

Download page also has search function so that users can quickly find out their interested data set for downloading. In addition, we also support the Application Programming Interface (API), which allows developers to obtain the analysis result from RPFdb by using a HTTP client. The server-side programs in RPFdb accept a fixed URL syntax for retrieval operations. For example, the search result of gene THI1 in Arabidopsis can be returned by using the URL http://sysbio.sysu.edu.cn/rpfdb/fetchExpression.php?gene=THI1&species=Arabidopsis. Besides, both gene symbol and Ensembl ID can be used as query keys.

### A case study

Here we show how to explore genes or studies of interest. For example, we want to know how mouse gene *Swi5* is translated under different conditions. On either the home page or the search page, we can select the species, mouse, and enter *Swi5* or Ensembl gene ID *ENSMUSG00000044627*. The output page displays RPKM of *Swi5* from 128 samples. We next want to know how *Swi5* is translated in mouse embryonic stem cells, ‘E14’. Entering ‘E14’ into the search box of the output page, it displays the results from the studies using ‘E14’ (11,17) (Supplemental Figure S5A). The output shows that RPKM in 3′ UTR is lower than that in 5′ UTR and CDS for all samples. And RPKM in 5′ UTR is much higher than that in CDS for the harringtonine-treated samples (Supplemental Figure S5A). This can be explained that ribosomes dropped after stop codon in 3′ UTR whereas ribosomes accumulate at initiation sites in 5′ UTR for the harringtonine treated cells (11). If we want to explore reads distribution of cycloheximide and harringtonine treated cells in the genome browser. We select samples of interest and click ‘Genome Browse’. The output page shows the reads coverage on genome (Supplemental Figure S5B). It shows that the harringonine-treat cells have one clear peak in the 5′ UTR region, in contrast, cycloheximide treated cells have reads in multiple exon regions and 5′ UTR region (Figure Supplemental Figure S5C and SD). These features are consistent with the results presented in the original publication (11).

Another useful function of RPFdb is to query studies of interest. For example, we enter ‘cell cycle’ in the search study page. The output page shows the relevant study of Stumpf CR *et al*. (18). Click ‘Details’, it leads to the page displaying the summary of the study and global perspective of the genome-wide distribution of RPF reads (Supplemental Figure S2).

## DISCUSSION

The numbers of studies using RPF technique have been growing significantly in the recent years. There is a strong need for an integrated database that facilitates the exploration of data from these studies. Here we present RPFdb, a comprehensive resource for hosting and analyzing the publicly available RPF data sets. The main functions of RPFdb include to search studies of interest, to explore basic statistics of reads of the studies, to compare reads of translated mRNA of given genes from multiple studies and to visualize RPF data in a genome browser.

GWIPS-viz (9) and RPFdb are both useful resources for users who are interested in translated mRNA but with little computational knowledge. In order to help users to choose an appropriate database, we compared RPFdb and GWIPS-viz regarding the number of studies, the type of collected data sets, data processing method, aligner, genome browser and main features (Table 1).

**Table 1.** Comparison of RPFdb and GWIPS-viz

	RPFdb	GWIPS-viz
No. of studies	82	45
Type of data sets	RPF only	RPF and mRNA-seq
Data processing	• The first 26 nucleotides kept;	• The adaptor linker sequence or poly-(A) tails trimmed from the 3′ ends of reads;
	• One mismatch allowed	• Three mismatches with alignment allowed
Aligner	STAR	Bowtie
Genome browser	Jbrowse	UCSC genome browser
Meta information	Searchable	Not searchable
Main features	• Statistics of studies and samples;	• Visualization of RPF, inferred A-sites and mRNA;
	• RPKM of RPF on different genomic location;	• Comparison of RPF and mRNA from the same sample
	• Visualization of RPF	

Owing to the increasing interest in ribosome profiling and in translational regulation in general, we envision that ribosome profiling technology will be applied to a broader set of species and conditions and more publications will be released in future. RPFdb will be updated in a timely manner with new released data from public studies. We will also make efforts to improve the database. We hope that RPFdb will be a valuable resource for both experimental and computational biologists who are interested in understanding translational regulation and gene regulation.
